# A new look at the decomposition of agricultural productivity growth incorporating weather effects

**DOI:** 10.1371/journal.pone.0192432

**Published:** 2018-02-21

**Authors:** Eric Njuki, Boris E. Bravo-Ureta, Christopher J. O’Donnell

**Affiliations:** 1 Department of Agricultural and Resource Economics, University of Connecticut, Storrs, Connecticut, United States of America; 2 University of Talca, Talca, Chile; 3 School of Economics, University of Queensland, St. Lucia, Queensland, Australia; Iowa State University, UNITED STATES

## Abstract

Random fluctuations in temperature and precipitation have substantial impacts on agricultural output. However, the contribution of these changing configurations in weather to total factor productivity (TFP) growth has not been addressed explicitly in econometric analyses. Thus, the key objective of this study is to quantify and to investigate the role of changing weather patterns in explaining yearly fluctuations in TFP. For this purpose, we define TFP to be a measure of total output divided by a measure of total input. We estimate a stochastic production frontier model using U.S. state-level agricultural data incorporating growing season temperature and precipitation, and intra-annual standard deviations of temperature and precipitation for the period 1960–2004. We use the estimated parameters of the model to compute a TFP index that has good axiomatic properties. We then decompose TFP growth in each state into weather effects, technological progress, technical efficiency, and scale-mix efficiency changes. This approach improves our understanding of the role of different components of TFP in agricultural productivity growth. We find that annual TFP growth averaged 1.56% between 1960 and 2004. Moreover, we observe substantial heterogeneity in weather effects across states and over time.

## Introduction

According to the United States National Climate Assessment: “Climate change poses a major challenge to U.S. agriculture because of the critical dependence of the agricultural system on climate and because of the complex role agriculture plays in rural and national social and economic systems” [1]. This challenge is of major concern given the critical role that this country plays in global food production and world food markets. It is noteworthy that in 2016, the U.S. generated approximately 35% of global corn supply, 33% of global soybeans and close to 33% of global dairy products [2]. Thus, understanding how to manage the agricultural sector in the face of climate change will enable the development of effective strategies aimed at coping with this challenge.

The central argument of this study is that climate change alters weather outcomes, and these outcomes have a direct biophysical effect on agricultural production [3]. Therefore, if agricultural output is impacted by weather outcomes then, *ceteris paribus*, total factor productivity (TFP) will also be affected. Following Dell et al. [4], we use the term *climate* to refer to the distribution of outcomes over long intervals (e.g., over several decades), while weather describes a particular realization from that distribution. Hence, *weather variation* refers to shorter-run temporal variation in temperature and precipitation within a given spatial area [4]. Thus, the key objective of this study is to investigate and to quantify the role of changing configurations in weather in explaining year-to-year fluctuations in TFP. By exploiting variations in weather outcomes one can identify their causal effects on agricultural productivity. In this sense, the maximal possible output in a given year is affected not only by the average temperature and precipitation experienced in that year, but also by within-year variations in temperature and precipitation.

Using data prepared by the Economic Research Service (ERS) of the U.S. Department of Agriculture for the period 1960–2004, this article estimates a stochastic production frontier that incorporates input-output data (i.e., total output, land, labor, capital, intermediate materials), weather variables (i.e., growing season temperature and precipitation, and intra-annual standard deviations of temperature and precipitation) and time-invariant characteristics of the production environment (e.g., topography). The ERS relied on the U.S. Bureau of Labor Statistics (BLS) to acquire a portion of the labor data that was used to develop the state-level agricultural data series. The BLS stopped collecting a key segment of the labor data in 2004. Subsequently, lacking some complementary or new data, the ERS stopped collection of state-level data in 2004. Since then, ERS has transitioned to national-level input-output data.

The estimated model is subsequently used to decompose a TFP index into four main components: weather effects, which capture fluctuations in TFP due to variations in temperature and precipitation; technological change, which measures shifts of the production frontier due to the discovery of new technologies; technical efficiency change, which measures movements towards or away from the frontier due to the use of different technologies; and scale efficiency changes, that measure productivity gains linked to economies of scale. Briefly, we find that annual TFP growth averaged 1.56% between 1960 and 2004. In addition, weather contributed to a 0.012% decline in annual TFP growth, on average, with considerable heterogeneity over time and space.

### Temperature and precipitation trends across the United States

**Fig 1:**
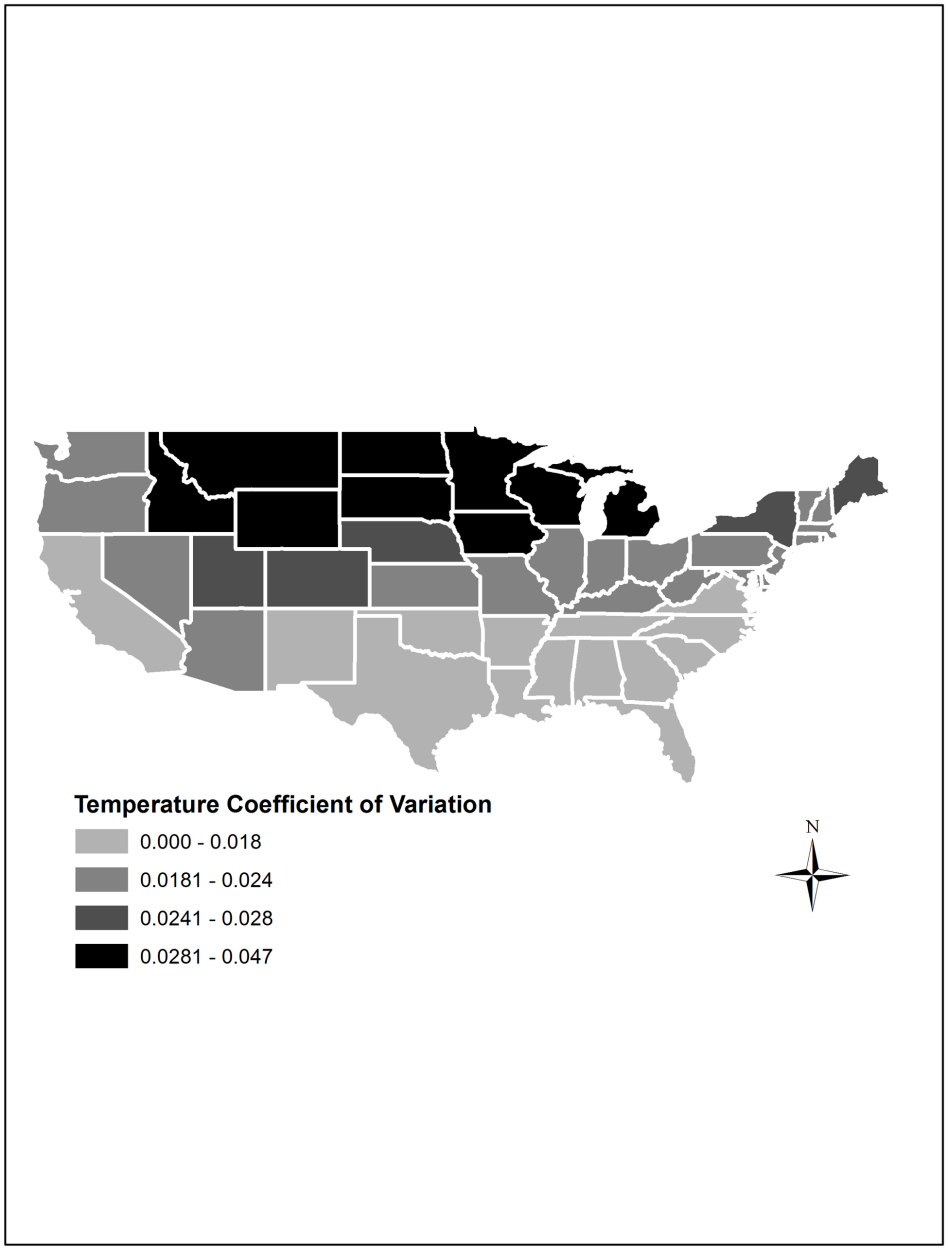
Year-to-year temperature coefficient of variation across the United States, 1960–2004.

Analyzing the shifting patterns of temperature and precipitation across the U.S. will enable us to gain an understanding of the potential effects of weather variability while underscoring the extent of anomalies and shocks in domestic weather patterns across various states. Estimates of the evolution of the spatial distribution of the coefficients of variation, calculated by dividing the year-to-year standard deviations of temperature and precipitation by their respective means, indicate that between 1960 and 2004, states in the northern part of the country (e.g., Idaho, Montana, Wyoming, North Dakota, South Dakota, Iowa, Minnesota, Wisconsin and Michigan) experienced substantial year-to-year temperature variability compared to the rest of the United States, as illustrated in Fig 1.

Note that Temperature scales that utilize Celsius and Fahrenheit measures are interval scales that can take on both positive and negative values. Thus, a measure of coefficient of variation for temperature where the mean is zero may lead to a value that is undefined. Furthermore, mean values close to zero may cause the coefficient of variation measure to explode. Consequently, in order to estimate the coefficient of variation for temperature, we use the Kelvin scale, which is a ratio scale that only takes non-negative values.

**Fig 2:**
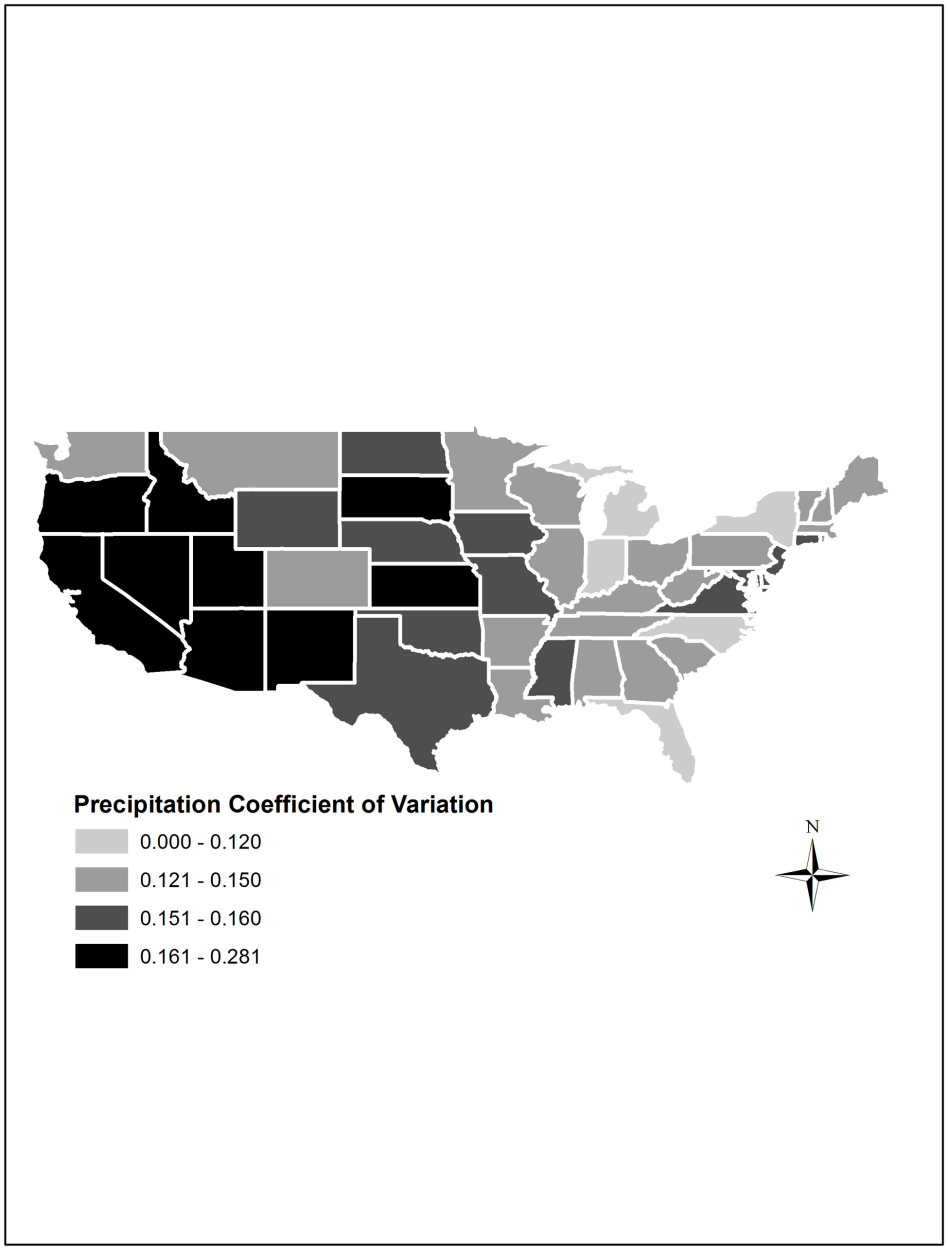
Year-to-year precipitation coefficient of variation across the United States, 1960–2004.

On the other hand, states in the west and southwest of the United States (e.g., Oregon, California, Idaho, Nevada, Utah, Arizona, New Mexico), and Kansas and South Dakota in the Northern Plains, were characterized by intense year-to-year precipitation variability, compared to the rest of the country, as depicted in Fig 2.

In sum, we observe considerable heterogeneity in the variability of temperature and precipitation across the U.S. over the period 1960–2004. The rest of this study will examine how this variability has impacted agricultural production patterns across the country.

### Climatic effects on U.S. agriculture

Early studies in economics focusing on climate change predicted significant mid-continental warming in the U.S. brought about by increased concentrations of atmospheric CO_2_ [5,6]. The seminal paper by Mendelsohn et al. [7] was one of the first to report quantitative estimates of the economic effects of climate change on U.S. agriculture. In contrast to earlier work, this latter study presented evidence that climate change might be beneficial to the U.S. farm sector as a whole. Mendelsohn and colleagues used a hedonic approach that specified land values as a function of climatic factors, and economic and demographic variables. Their analysis predicted regional adjustments in crop and livestock production, as well as in resource use in response to climate change. However, the Mendelsohn and coauthors approach was criticized in the literature for applying cross-sectional data while implicitly assuming a perfectly elastic supply of irrigation water [8], and for overstating the potential benefits of warmer weather [9]. Moreover, Kaufman [10] questioned the stability of the regression coefficients reported and argued that this undermined the credibility of their results.

Several studies have followed in the footsteps of Mendelsohn et al. [7], including Adams et al. [11], Mendelsohn and Dinar [12], Lobell and Asner [13], Schlenker et al. [14,15], Deschenes and Greenstone [16], Roberts et al. [17], and Burke and Emerick [18]. Some of these studies evaluate the responsiveness of profit-maximizing economic agents to changing configurations in weather; common responses include switching to more drought resistant crops or the adoption of improved irrigation systems [16]. The results of these studies have yielded a wide range of predicted impacts on U.S. agriculture. Some studies have predicted gains in U.S. agriculture due to climate variability [11,16], declining agricultural yields [13,18], while others anticipate mild impacts [12] or a mixture of results [19]. A key argument is that U.S. agricultural production will be impacted primarily due to changing configurations of temperature and precipitation, and this will lead directly to modifications in farming patterns and resource use [7], and a growing reliance on secondary sources for water such as irrigation [12,14]. The implementation of adaptation strategies can be expected to reduce the long-run adverse effects stemming from changes in climatic conditions [14].

In contrast to the hedonic studies aforementioned, this study uses a stochastic production frontier approach to examine the direct impact of changing configurations in weather on agricultural output, and therefore TFP. As noted earlier, isolating the role of changing weather patterns on TFP growth in the U.S. agricultural sector has for the most part been ignored; hence, this article seeks to address this gap in the literature. The rest of the study is organized as follows: The next section presents the theoretical foundation that models the production technology, as well as the general index that is used to measure and decompose TFP, followed by a discussion of the data and econometric specification. We then present the results, and finally, a summary and concluding remarks.

## The production technology

This section presents the methodology that is used to characterize the production technology. This article distinguishes between the production technology and environmental factors that impact production outcomes. To be specific, a technology is defined as “…a technique, method or system for transforming inputs into outputs” [20]. On the other hand, environmental factors consist of all exogenous variables that are physically involved in the production process but that are beyond the control of the firm. In the context of this article, the environmental factors of relevance are weather variables and time-invariant regional features such as topography. The set of all technologies available in period *t* is referred to as the period-*t* technology set. In addition, the set of all input-output combinations that are feasible using a given technology set in a given period in a given environment is referred to as a period-and-environment-specific production possibilities set. For example, in mathematical terms, the set of output-input combinations that can be produced using the period-*t* technology set in environment *z* is given as:

$$T^{t}\left(z\right)=\left\{\left(x,q\right)\in {ℜ}_{+}^{M+N}:x\;\text{can}\;\text{produce}\;q\;\text{in}\;\text{environment}\;z\;\text{in}\;\text{period}\;t\right\}$$ (1)

Following O’Donnell (2016), we assume the following properties: (1) (*x*, 0) ∈ *T*^*t*^ (*z*) for any $x\in R_{+}^{M}$ implying that inactivity is possible; (2) the output set *P*^*t*^ (*x*, *z*) ≡ {*q*: (*x*, *q*) ∈ *T*^*t*^ (*z*)} is bounded for all $x\in R_{+}^{M}$; (3) if *q* > 0, then (0, *q*) ∉ *T*^*t*^ (*z*), implying that a strictly positive amount of at least one input is required to produce a positive amount of output; (4) if (*x*, *q*) ∈ *T*^*t*^ (*z*) and 0 ≤ *λ* ≤ 1, then (*x*, *λq*) ∈ *T*^*t*^ (*z*), implying outputs are weakly disposable; (5) the output set *P*^*t*^ (*x*, *z*) ≡ {*q*: (*x*, *q*) ∈ *T*^*t*^ (*z*)} is closed, implying the set of outputs that can be produced given an input vector contains all the points on its boundary. If these properties are satisfied, then the period-*t* technology set can be represented using period-and-environment-specific distance functions. The period-and-environment-specific output distance function (ODF) and input distance function (IDF) are defined as $D_{O}^{t}(x,q,z)=inf\{\rho >0:(x,q/\rho )\in T^{t}(z)\}$ and $D_{I}^{t}(x,q,z)=sup\{\theta >0:(x/\theta ,q)\in T^{t}(z)\}$. By construction, the ODF is nonnegative and homogeneous of degree one in outputs. The IDF is nonnegative and homogeneous of degree one in inputs.

In addition to the properties listed above, we also assume (6) if (*x*, *q*) ∈ *T*^*t*^ (*z*) and $0\leq \tilde{q}\leq q$, then $\left(x,\tilde{q})\in T^{t}(z\right)$, i.e., outputs are strongly disposable. Strong disposability of outputs implies that it is possible to use the same vector of inputs to produce fewer outputs. This guarantees that the ODF is non-decreasing in outputs. Finally, we assume (7) if (*x*, *q*) ∈ *T*^*t*^ (*z*) and *λ* > 0, then (*λx*, *λ*^*r*^*q*) ∈ *T*^*t*^ (*z*), which implies that the technology set is homogeneous of degree *r*. This implies the ODF is homogeneous of degree–*r* in inputs, the IDF is homogeneous of degree –1/*r* in outputs, and $-(1/r)\;\text{ln}\;D_{O}^{t}(x,q,z)=\text{ln}\;D_{I}^{t}(x,q,z)$ (e.g., O’Donnell, 2016, Proposition 3).

### An axiomatic approach to TFP decomposition

The TFP index that is used in this article is the general index proposed in O’Donnell [20]. This is an economic-theoretic index that is proper in the sense that it satisfies several basic axioms, including monotonicity, linear homogeneity, identity, commensurability, proportionality, and transitivity. In contrast, the widely used Fisher, Tornqvist, and Malmquist productivity indexes do not satisfy the transitivity axiom, which is a critical property when making comparisons across data points [21–28]. The transitivity axiom states that a direct comparison of the TFP of two decision-making units (DMUs) should yield the same estimate of TFP change as an indirect comparison through a third DMU [29].

The general index of O’Donnell is obtained by dividing a general output quantity index by a general input quantity index. These indexes are general in the sense that they nest several other proper indexes as special cases. For example, if there are no environmental variables in the production process and there is no technical change, then the general output and input quantity indexes collapse to indexes found in Färe and Primont [30]. Furthermore, like any proper TFP index, the general TFP index can be decomposed into measures of environmental change, technological progress, and technical, scale, and mix efficiency changes [20].

#### Total factor productivity change

We begin this section by introducing *i* and *t* subscripts into the notation so that, for example, *q*_*it*_ now denotes the outputs of state *i* in period *t*. We define total factor productivity (TFP) as the ratio of an aggregate output to an aggregate input. More formally, the TFP of state *i* in year *t* is:

$$TFP_{it}=\frac{Q_{it}}{X_{it}}$$ (2)

where *Q*_*it*_ ≡ *Q* (*q*_*it*_) is an aggregate output, *X*_*it*_ ≡ *X* (*x*_*it*_) is an aggregate input, and *Q*(.) and *X*(.) are nonnegative, non-decreasing and linearly homogeneous scalar functions. If outputs and inputs are strongly disposable, as we assume in this paper, then possible aggregator functions are $Q(q)=D_{O}^{\overset{-}{t}}(\overset{-}{x},q,\overset{-}{z})$ and $X(x)=D_{I}^{\overset{-}{t}}(x,\overset{-}{q},\overset{-}{z})$, where $\overset{-}{x}$, $\overset{-}{q}$ and $\overset{-}{z}$ are respectively fixed vectors of inputs, outputs and environmental variables, and $\overset{-}{t}$ is a fixed reference period. The associated index that compares the TFP of state *i* in year *t* with the TFP of state *k* in year *s* is the general index defined [20] as:

$$TFPI_{ksit}^{G}=\frac{D_{O}^{\bar{t}}\left(\bar{x},q_{it},\bar{z}\right)}{D_{O}^{\bar{s}}\left(\bar{x},q_{ks},\bar{z}\right)}\frac{D_{I}^{\bar{s}}\left(x_{ks},\bar{q},\bar{z}\right)}{D_{I}^{\bar{t}}\left(x_{it},\bar{q},\bar{z}\right)}.$$ (3)

Special cases of (3) include the Lowe, geometric Young and Färe-Primont TFP indexes (TFPIs). The mathematical form of the general TFPI in (3) depends on the mathematical form of the output and input distance functions. For example, suppose there is only one output and the log-distance function is given by the following Cobb-Douglas (CD) function:

$$\text{ln}D_{O}^{t}\left(x_{it},q_{it},z_{it}\right)=\text{ln}q_{it}-\phi_{i}-\overset{H}{\underset{h=1}{\sum }}\gamma_{h}d_{hit}t-\overset{J}{\underset{j=1}{\sum }}\rho_{j}\text{ln}z_{jit}-\overset{M}{\underset{m=1}{\sum }}\beta_{m}\text{ln}x_{mit}$$ (4)

where *ϕ*_*i*_ is a fixed environmental effect and *d*_*hit*_ is a dummy variable that takes the value 1 if period *t* is in the *h*-th quinquennium (and 0 otherwise). In this case, the TFPI defined by expression (3) takes the form:

$$TFPI_{ksit}^{G}=\frac{q_{it}}{q_{ks}}\left[\overset{M}{\underset{m=1}{\prod }}{\left(\frac{x_{mks}}{x_{mit}}\right)}^{\lambda_{m}}\right]$$ (5)

where *λ*_*m*_ = *β*_*m*_/*r* and *r* = ∑_*m*_ *β*_*m*_ is the elasticity of scale. Computing this index obviously involves estimating *β*_1_,…, *β*_*M*_. In this article, we estimate these parameters using stochastic frontier analysis (SFA) methods.

## Data and econometric specification

The data used consists of indices of farm output and inputs across the 48 contiguous states of the U.S. and is developed by the Economic Research Service (ERS) of the U.S. Department of Agriculture [31]. Several authors have used similar data to analyze different productivity issues [20,27,29,32,33]. The aggregate output index is constructed by the ERS from measures of physical quantities of livestock, crop and other outputs, and their respective state-level market prices. The input indices consist of land, labor, capital and intermediate materials, all calculated by the ERS. Details concerning the construction of the input and output indices are elaborated in Ball et al. [27,33,34]. This article utilizes the full data set for the 45-year period between 1960 and 2004; thus, the total number of observations is 2,160. Table 1 provides a summary of the descriptive statistics of the variables used in the stochastic production frontier analysis. The complete dataset used in this analysis is included as S1 File.

**Table 1:** Descriptive statistics of variables used in the stochastic production frontier model.

Variable	Observation	Mean	Std.Dev	Min	Max
Output	2160	1.14	1.16	0.01	9.33
Land	2160	2.1	2.23	0.01	15.12
Labor	2160	2.62	2.31	0.02	12.59
Capital	2160	1.87	1.67	0.02	9.41
Intermediate	2160	0.89	0.82	0.01	4.75
Temperature (Fahrenheit)	2160	52	7.63	36.53	72.58
Precipitation (mm)	2160	76.64	31.67	11.37	170.56
Intra-annual Temperature	2160	4.48	2.76	0.99	24.82
Intra-annual Precipitation	2160	33.27	14.51	4.76	88.02

The evidence pointing to the importance that changing weather patterns can have on agricultural production has been building [1,3,4,35,36]. Recent empirical work based on U.S. agricultural data has generated additional evidence at the farm and county levels [37–40]. Therefore, the ERS data is augmented with state-level averages of temperature and cumulative precipitation obtained from the National Centers for Environmental Information (NCEI) of the National Oceanic and Atmospheric Administration (NOAA). The NCEI collects data on temperature and precipitation values from approximately 10,000 stations distributed across the country. We rely on daily estimates of temperature and precipitation derived from the NCEI to construct state-level average growing season temperature, and cumulative precipitation measures.

Furthermore, the maximal possible output in a given year is affected not only by averages of growing season temperature and cumulative precipitation, but also by the variations in temperature and precipitation within that season. For example, in any given growing season, temperatures above the norm would be expected to negatively impact farm output. Similarly, if all the precipitation falls in a few episodes, or not at all, then we would expect an adverse effect on output compared to a situation where the rain is spread more evenly throughout the growing season. Thus, in order to capture these shocks and anomalies over the growing season we use estimates of intra-annual (within-year) standard deviations calculated from average daily temperature and precipitation measures. These are then averaged within each individual State. This specification is consistent with a number of studies including Mendelsohn et al. [41], Lobell et al. [13] and Kaminski et al. [42]. Temperature and precipitation measures in the U.S. are highly seasonal which makes it difficult to identify long-term trends. We therefore adjust these measures for seasonality using local regression (LOESS) procedures [43] in order to identify trends over time.

### Specification of the stochastic production frontier

The Cobb-Douglas specification is selected over other functional forms (e.g., the translog) because it enables us to obtain a neat decomposition of the sources of productivity growth as outlined in Eq (8) below. Our Cobb-Douglas stochastic production frontier model is:

$$\text{ln}q_{it}=\phi_{i}+\sum_{h=1}^{H}\gamma_{h}d_{hit}t+\sum_{j=1}^{J}\rho_{j}\text{ln}z_{jit}+\sum_{m=1}^{M}\beta_{m}\text{ln}x_{mit}+v_{it}-u_{it}$$ (6)

where *ϕ*_*i*_ is an intercept that varies across each state and is meant to capture unobserved heterogeneity (e.g., time-invariant features of the environment, such as topography); *d*_*hit*_ are dummy variables that allow the rate of technical change to vary across quinquennials; *x*_1*it*_,…, *x*_*Mit*_ are conventional inputs (i.e., land, labor, capital, intermediate inputs); *z*_1*it*_,…, *z*_*Jit*_ are weather variables broken into two sets comprising growing season averages of temperature and cumulative precipitation, as well as intra-annual standard deviations of temperature and precipitation that capture shocks and anomalies in the weather patterns within a given year; *v*_*it*_ is an unobserved variable accounting for functional form errors (e.g., the possibility that the true production frontier is not CD), measurement errors (e.g., errors in the measurement of total output), and other sources of statistical noise (e.g., omitted variable errors); and $u_{it}=-\text{ln}D_{O}^{t}(x_{it},q_{it},z_{it})$ is a nonnegative technical efficiency effect. If there is no statistical noise (i.e., if *v*_*it*_ = 0), then Eq (6) is equivalent to Eq (4). We make the common assumption that the noise and inefficiency effects are independent random variables with $v_{it}\sim N(0,\sigma_{v}^{2})$ and $u_{it}\sim N^{+}(0,\sigma_{u}^{2})$. It is noteworthy to point out that by incorporating state-level fixed effects, the specification given by Eq (6) has the same statistical structure as the true fixed effects model of Greene [44,45].

#### Decomposing TFP change

The stochastic production frontier model given by Eq (6) can be used to decompose the general index defined by Eq (5). An easy way to do this is to take the antilogarithm of Eq (6) and write

$$q_{it}=\text{exp}\left(\sum_{h=1}^{H}\gamma_{h}d_{hit}t\right)\left[\text{exp}(\phi_{i})\prod_{j=1}^{J}z_{jit}{}^{\rho_{j}}\right]\left[\prod_{m=1}^{M}x_{mit}{}^{\beta_{m}}\right]\text{exp}\left(-u_{it}\right)\;\text{exp}(v_{it}).$$ (7)

This equation can be used to substitute *q*_*it*_ and *q*_*ks*_ out of Eq (5), resulting in the following decomposition of TFP change:

$$TFPI_{ksit}^{G}=\left[\frac{\text{exp}\left(\sum_{h=1}^{H}\gamma_{h}d_{hit}t\right)}{\text{exp}\left(\sum_{h=1}^{H}\gamma_{h}d_{ks}s\right)}\right]\left[\frac{\text{exp}\left(\phi_{i}\right)}{\text{exp}\left(\phi_{k}\right)}\overset{J}{\underset{j=1}{\prod }}{\left(\frac{z_{jit}}{z_{jks}}\right)}^{\rho_{j}}\right]\left[\overset{M}{\underset{m=1}{\prod }}{\left(\frac{x_{mit}}{x_{mks}}\right)}^{\beta_{m}-\lambda_{m}}\right]\left[e^{\left(u_{ks}-u_{it}\right)}\right]\left[e^{\left(v_{ks}-v_{it}\right)}\right]$$ (8)

The first term on the right-hand side is an output-oriented technology index (OTI), i.e., a measure of technological progress; the second term is an output-oriented environment index (OEI), i.e., a measure of changes in fixed characteristics of the environment (e.g, topography) and weather; the third term is an output-oriented scale efficiency index (OSEI) i.e., a measure of changes in economies of scale (if *r* = 1, then the technology exhibits constant returns to scale and this last component drops out); the fourth term is an output-oriented technical efficiency index (OTEI), i.e., a measure of movements towards or away from the frontier; and the last term is a statistical noise index (SNI) (i.e., a measure of changes in statistical noise). Kneip and Sickles [46] argue that decomposing TFP change, even with elaborate econometric formulations, is a challenging task and hence some statistical noise is likely to remain.

## Results

Prior to discussing our results, we acknowledge possible concerns regarding the potential for endogeneity in stochastic production frontier models [47–49]. A possible source of endogeneity is that input choices may be driven by weather outcomes. Verbeek [50] and O’Donnell [20] claim that if at least one of the explanatory variables is an I(1) process and the dependent and explanatory variables are cointegrated, then least squares estimators for the slope parameters will be super-consistent even if some of the variables are endogenous. We tested for unit roots using the panel unit root test of Maddala and Wu [51]. Using 4 lags, we failed to reject the null hypothesis of a unit root at the 5% level of significance for the dependent variable, and the explanatory variables; land, labor, irrigation, precipitation and temperature. We then conducted a Pedroni [52] test and concluded that the variables are cointegrated. Maximum likelihood estimates of the parameters of the stochastic production frontier model are provided in Table 2.

**Table 2:** Coefficient estimates of stochastic production frontier model.

Variable		Coeff. Estimates	(Standard Errors)
Land	β_1_	0.1044***	(0.0179)
Labor	β_2_	0.1065***	(0.0096)
Capital	β_3_	0.1018***	(0.0188)
Intermediate	β_4_	0.5628***	(0.0130)
Temperature	ρ_1_	-0.4259***	(0.0940)
Precipitation	ρ_2_	0.0261***	(0.0087)
Intra-annual Temp.	ρ_3_	0.0031	(0.0049)
Intra-annual Prec.	ρ_4_	-0.0131**	(0.0062)
Q1 (1960–64)	γ_1_	0.0094***	(0.0030)
Q2 (1965–69)	γ_2_	0.0072***	(0.0014)
Q3 (1970–74)	γ_3_	0.0082***	(0.0010)
Q4 (1975–79)	γ_4_	0.0082***	(0.0008)
Q5 (1980–84)	γ_5_	0.0094***	(0.0006)
Q6 (1985–89)	γ_6_	0.0118***	(0.0005)
Q7 (1990–94)	γ_7_	0.0126***	(0.0005)
Q8 (1995–99)	γ_8_	0.0118***	(0.0004)
Q9 (2000–04)	γ_9_	0.0119***	(0.0004)
AL	ϕ_1_	1.3977***	(0.4223)
AR	ϕ_2_	1.4512***	(0.4185)
AZ	ϕ_3_	1.5411***	(0.4215)
CA	ϕ_4_	2.0849***	(0.4115)
CO	ϕ_5_	1.2509***	(0.3990)
CT	ϕ_6_	1.1868***	(0.4048)
DE	ϕ_7_	1.3108***	(0.4127)
FL	ϕ_8_	1.9254***	(0.4292)
GA	ϕ_9_	1.6162***	(0.4224)
ID	ϕ_10_	1.4156***	(0.3949)
IL	ϕ_11_	1.6087***	(0.4149)
IN	ϕ_12_	1.4607***	(0.4122)
IA	ϕ_13_	1.6321***	(0.4110)
KS	ϕ_14_	1.4294***	(0.4183)
KY	ϕ_15_	1.4201***	(0.4159)
LA	ϕ_16_	1.2977***	(0.4259)
ME	ϕ_17_	1.1249***	(0.3941)
MD	ϕ_18_	1.2589***	(0.4120)
MA	ϕ_19_	1.2823***	(0.4027)
MI	ϕ_20_	1.3064***	(0.4009)
MN	ϕ_21_	1.4328***	(0.4025)
MS	ϕ_22_	1.4099***	(0.4238)
MO	ϕ_23_	1.3529***	(0.4174)
MT	ϕ_24_	1.0620***	(0.3993)
NE	ϕ_25_	1.4387***	(0.4108)
NV	ϕ_26_	0.9692**	(0.4022)
NH	ϕ_27_	0.9091**	(0.3990)
NJ	ϕ_28_	1.3630***	(0.4106)
NM	ϕ_29_	1.0693***	(0.4123)
NY	ϕ_30_	1.4206***	(0.4015)
NC	ϕ_31_	1.7082***	(0.4175)
ND	ϕ_32_	1.2398***	(0.4016)
OH	ϕ_33_	1.4287***	(0.4103)
OK	ϕ_34_	1.2885***	(0.4232)
OR	ϕ_35_	1.3408***	(0.3971)
PA	ϕ_36_	1.3711***	(0.4061)
RI	ϕ_37_	1.0647***	(0.4073)
SC	ϕ_38_	1.4017***	(0.4212)
SD	ϕ_39_	1.2609***	(0.4069)
TN	ϕ_40_	1.2689***	(0.4176)
TX	ϕ_41_	1.4454***	(0.4302)
UT	ϕ_42_	1.0856***	(0.4025)
VT	ϕ_43_	1.1180***	(0.3977)
VA	ϕ_44_	1.3207***	(0.4135)
WA	ϕ_45_	1.5696***	(0.3980)
WV	ϕ_46_	0.7804*	(0.4088)
WI	ϕ_47_	1.4269***	(0.4025)
WY	ϕ_48_	0.7910**	(0.3966)
Sigma (u_it_)	σ_uit_	0.0823	
Sigma (v_it_)	σ_vit_	0.0546	

Note:

***, significance at 1%.

**, significance at 5%.

* significance at 10%.

The coefficients for land, labor, capital and intermediate materials, interpreted as partial output elasticities, are nonnegative, which is consistent with our strong disposability assumption. A Wald test for the null hypothesis of constant returns to scale yields a test statistic of 461 with a p-value of 0.000. We therefore reject the null hypothesis that this model exhibits constant returns to scale. In fact, the sum of the coefficients indicates that the estimated elasticity of scale is $\hat{r}=0.8755,$ revealing decreasing returns to scale. A Wald test to check for the significance of including all the state-level fixed effects in the model yields a test statistic of 97.51 with a *p*-value of 0.000. Hence, we conclude that the state-level fixed effects belong in the model.

The parameter estimates for the weather variables reveal that higher values of growing-season temperature and precipitation, and the variability of precipitation (i.e., intra-annual precipitation) have statistically significant impacts on agricultural output. The way to interpret these parameters is that, *ceteris paribus*, a one percent increase in average growing season temperature leads to a statistically significant 0.426% reduction in output. Similarly, a one percent increase in intra-annual standard deviation of precipitation leads to a statistically significant 0.013% decline in agricultural output. Conversely, a one percent increase in precipitation leads to, *ceteris paribus*, a 0.026% increase in agricultural output.

Furthermore, instead of using a simple time-trend, which is restrictive because it assumes a constant rate of technological change across the entire sample period, our model allows the rate of technological change to vary quinquenially (i.e., every 5 years). The results indicate that technological change averaged 0.94% per annum between 1960–64, peaked at 1.26% per annum between 1990–94, before declining to 1.19% per annum over the 2000–04 period.

### Total factor productivity growth

**Fig 3:**
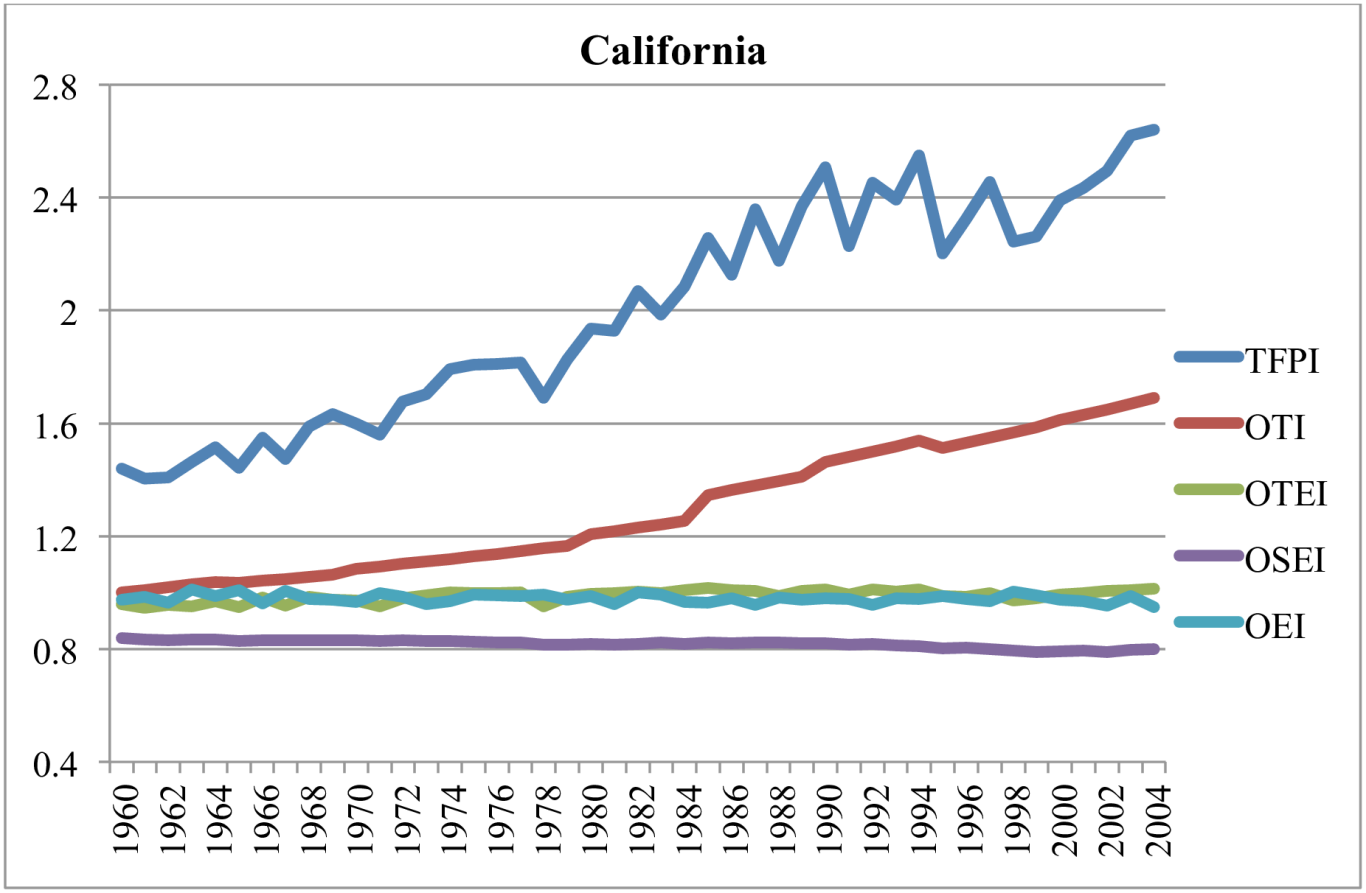
Estimates of TFPI and efficiency components in California, 1960–2004 (cf. AL in 1960).

**Fig 4:**
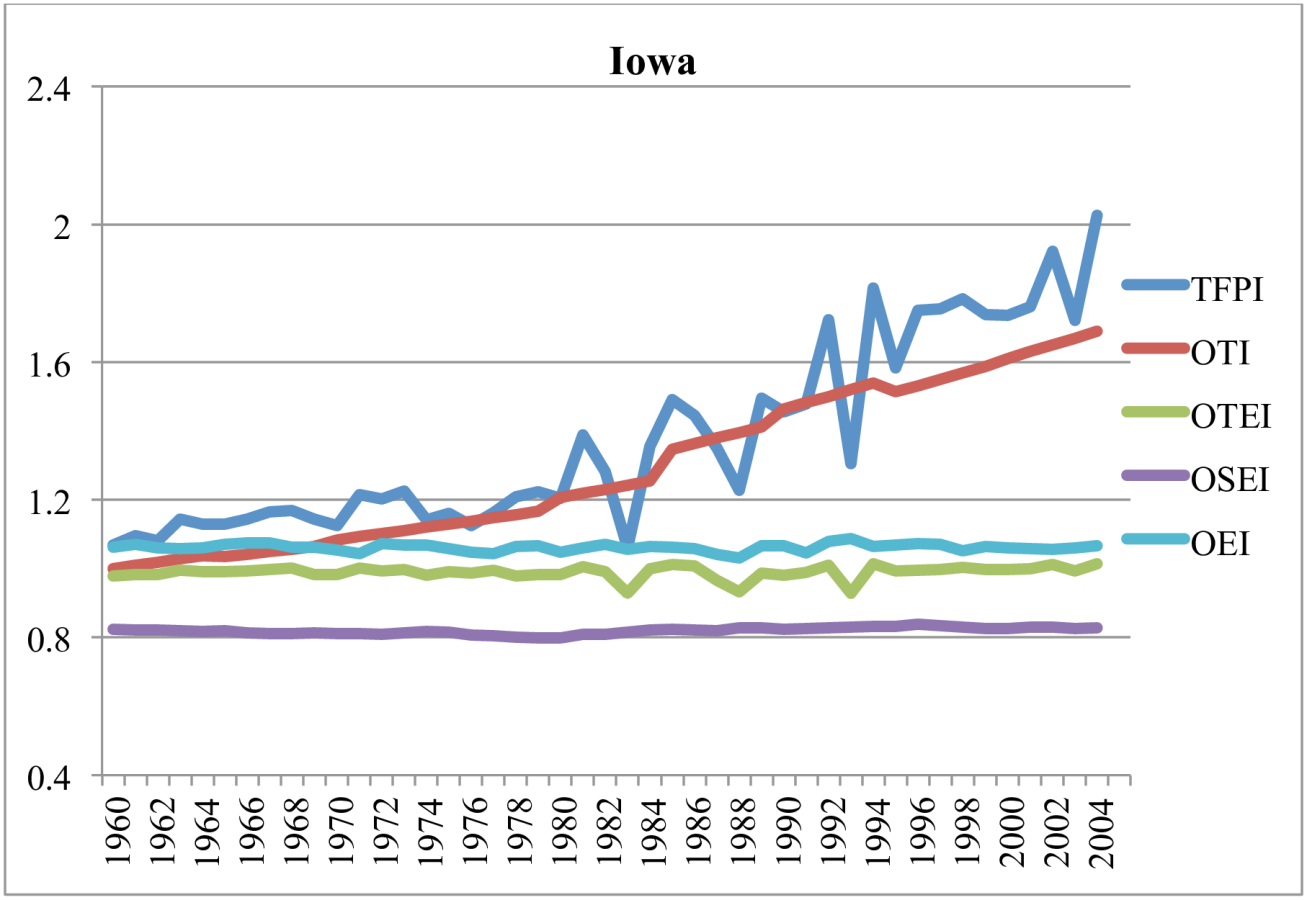
Estimates of TFPI and efficiency components in Iowa, 1960–2004 (cf. AL in 1960).

**Fig 5:**
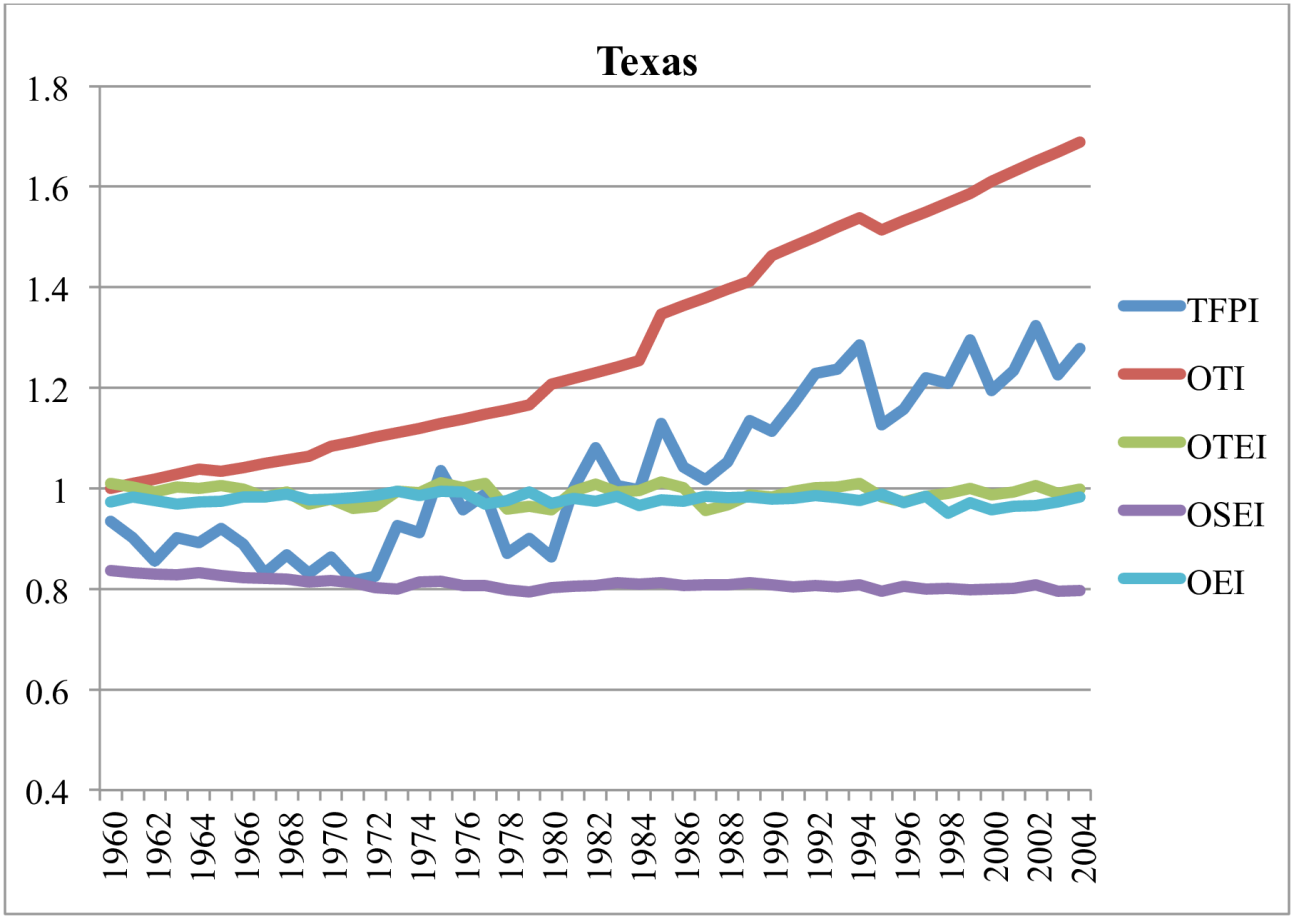
Estimates of TFPI and efficiency components in Texas, 1960–2004 (cf. AL in 1960).

**Fig 6:**
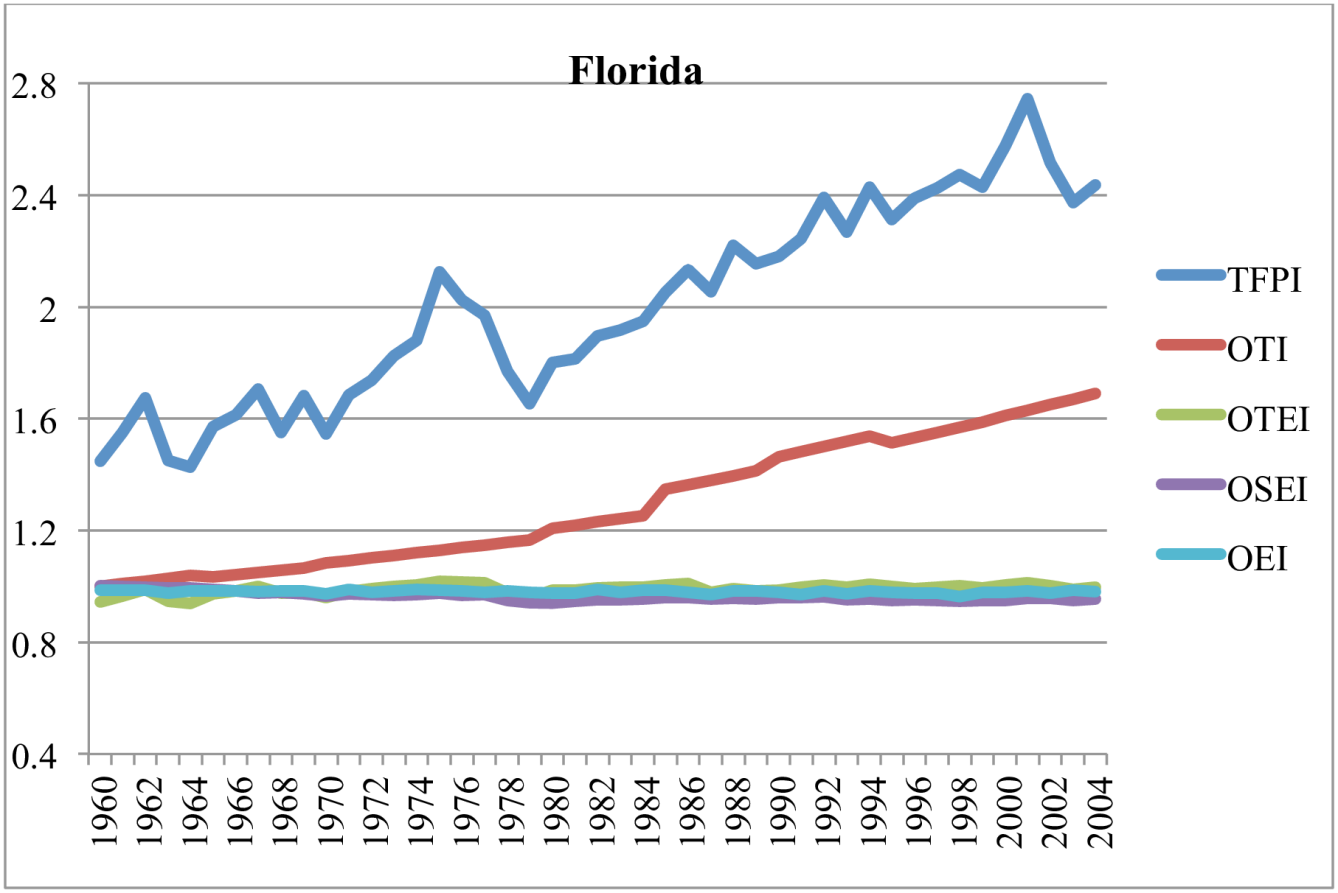
Estimates of TFPI and efficiency components in Florida, 1960–2004 (cf. AL in 1960).

Figs 3–6 illustrate components of the general TFP index (TFPI) for selected states: California, Iowa, Texas and Florida, between 1960 and 2004. All indexes compare the relevant variable in a particular year with the value of that variable in Alabama (AL) in 1960. In these figures, the components of the TFPI are the output-oriented technology index (OTI), output-oriented environment index (OEI), output-oriented technical efficiency index (OTEI) and output-oriented scale efficiency index (OSEI) in Eq (8).

Figs 3–6 reveal that TFP has been expanding, and that long-term growth in TFP has largely been driven by technological progress. The prominence of technological progress as a long-term driver of agricultural productivity is consistent with several other studies [53–57]. Hatfield et al. [1] posit that in the short-term technological advancements may mitigate a significant amount of negative impacts due to unfavorable weather.

Table 3 presents year-to-year TFP growth rates and its components for the U.S. and each of the 48 contiguous states for the period 1960–2004. The year-to-year TFP growth rate decompose as %ΔTFPI = %ΔOTI + %ΔOTEI + %ΔOSEI + %ΔOEI + %ΔSNI, where the right-hand-side components are percentage rates of growth in the indexes in Eq (8).

**Table 3:** Average annual growth rates TFP, 1960–2004.

STATE	%ΔTFPI	%ΔOTI	%ΔOTEI	%ΔOSEI	%ΔOEI	%ΔSNI
**U.S.**	**1.557**	**1.206**	**0.057**	**-0.085**	**-0.012**	**0.391**
AL	1.256	1.206	0.007	-0.228	0.005	0.267
AR	2.003	1.206	0.178	-0.346	-0.089	1.054
AZ	1.479	1.206	0.068	-0.063	-0.070	0.338
CA	1.560	1.206	0.142	-0.255	-0.048	0.515
CO	1.112	1.206	0.014	-0.226	0.053	0.065
CT	1.942	1.206	0.067	0.227	-0.039	0.481
DE	2.115	1.206	0.219	-0.238	-0.021	0.950
FL	1.379	1.206	0.132	-0.234	-0.015	0.290
GA	1.942	1.206	0.169	-0.212	-0.080	0.859
IA	2.035	1.206	0.121	-0.009	-0.021	0.738
ID	1.711	1.206	0.135	-0.246	0.003	0.613
IL	2.515	1.206	0.146	0.017	-0.082	1.228
IN	2.493	1.206	0.157	0.016	0.006	1.109
KS	1.091	1.206	-0.009	-0.275	0.046	0.123
KY	1.102	1.206	-0.075	-0.233	0.028	0.176
LA	1.605	1.206	0.083	-0.163	-0.010	0.489
MA	1.648	1.206	0.021	0.381	-0.065	0.105
MD	1.781	1.206	0.096	-0.062	-0.013	0.555
ME	1.984	1.206	0.087	0.328	-0.036	0.399
MI	1.709	1.206	0.090	-0.052	-0.094	0.559
MN	1.744	1.206	0.066	-0.125	-0.030	0.627
MO	1.705	1.206	0.044	-0.058	0.001	0.512
MS	1.585	1.206	0.042	-0.220	-0.026	0.583
MT	2.257	1.206	0.133	-0.039	0.005	0.952
NC	1.053	1.206	-0.042	-0.366	0.000	0.255
ND	3.100	1.206	0.294	-0.158	0.006	1.753
NE	1.508	1.206	0.079	-0.304	-0.029	0.557
NH	1.914	1.206	0.069	0.382	0.004	0.253
NJ	1.444	1.206	0.017	0.303	0.009	-0.090
NM	1.210	1.206	0.049	-0.300	-0.014	0.270
NV	1.168	1.206	0.034	-0.221	-0.036	0.186
NY	1.151	1.206	-0.020	0.129	0.003	-0.167
OH	1.723	1.206	0.054	-0.019	0.004	0.478
OK	0.353	1.206	-0.107	-0.300	0.014	-0.460
OR	2.278	1.206	0.213	-0.063	-0.004	0.927
PA	1.486	1.206	0.059	-0.053	-0.021	0.296
RI	2.177	1.206	0.092	0.450	-0.004	0.433
SC	1.468	1.206	-0.024	-0.140	0.008	0.418
SD	1.579	1.206	0.061	-0.168	0.024	0.456
TN	0.782	1.206	-0.157	-0.153	0.027	-0.141
TX	0.933	1.206	-0.012	-0.249	-0.028	0.017
UT	1.252	1.206	0.041	-0.121	0.037	0.089
VA	0.988	1.206	-0.060	-0.155	0.018	-0.021
VT	1.306	1.206	-0.017	0.143	-0.026	0.000
WA	1.717	1.206	0.207	-0.249	-0.038	0.592
WI	1.071	1.206	-0.046	-0.033	-0.009	-0.046
WV	0.715	1.206	-0.063	0.037	0.008	-0.473
WY	0.615	1.206	-0.114	-0.159	0.062	-0.380

We find that the TFP growth rate averaged 1.56% per annum across the United States. We also find that technological progress and output-oriented technical efficiency contributed to an average increase of 1.21% and 0.057% per annum in TFP, whereas environmental effects and output-oriented scale efficiency contributed to an average decline of 0.012% and 0.085% per annum in TFP, respectively. The TFP growth rates range from a high of 3.1% per annum in North Dakota (ND) to a low of 0.35% per annum in Oklahoma (OK). In comparison, Ball et al. [32] found a 1.94% TFP annual growth rate during the period 1948 to 1994 for the U.S. Findings from Jorgenson et al. [58] reveal a 1.90% TFP growth rate in U.S. agriculture for the period 1977 to 2000. O’Donnell [59] finds a growth rate of approximately 1.68% for the period 1960–2004. In a recent study, Ball et al. [33] report a 1.74% average growth rate over the period 1960 to 2004. These differences in TFP growth rates are due to differences in the TFP indices. Moreover, some of the aforementioned studies use indices that are not transitive, and therefore they are not reliable measures of TFP change. The index used here is transitive, as is the index used by O’Donnell [59]. The difference between the growth rates reported here and those reported in O’Donnell [59] is due to the fact that the output and input indices used here are ratios of weighted geometric averages, whereas the indices in O’Donnell [59] are ratios of weighted arithmetic averages. It is worth pointing out that our results are the only ones that unambiguously incorporate weather variation and other agro-ecological conditions, state-level fixed effects, and quinquennial time-effects in the model and subsequent decomposition of TFP.

#### Weather effects

**Fig 7:**
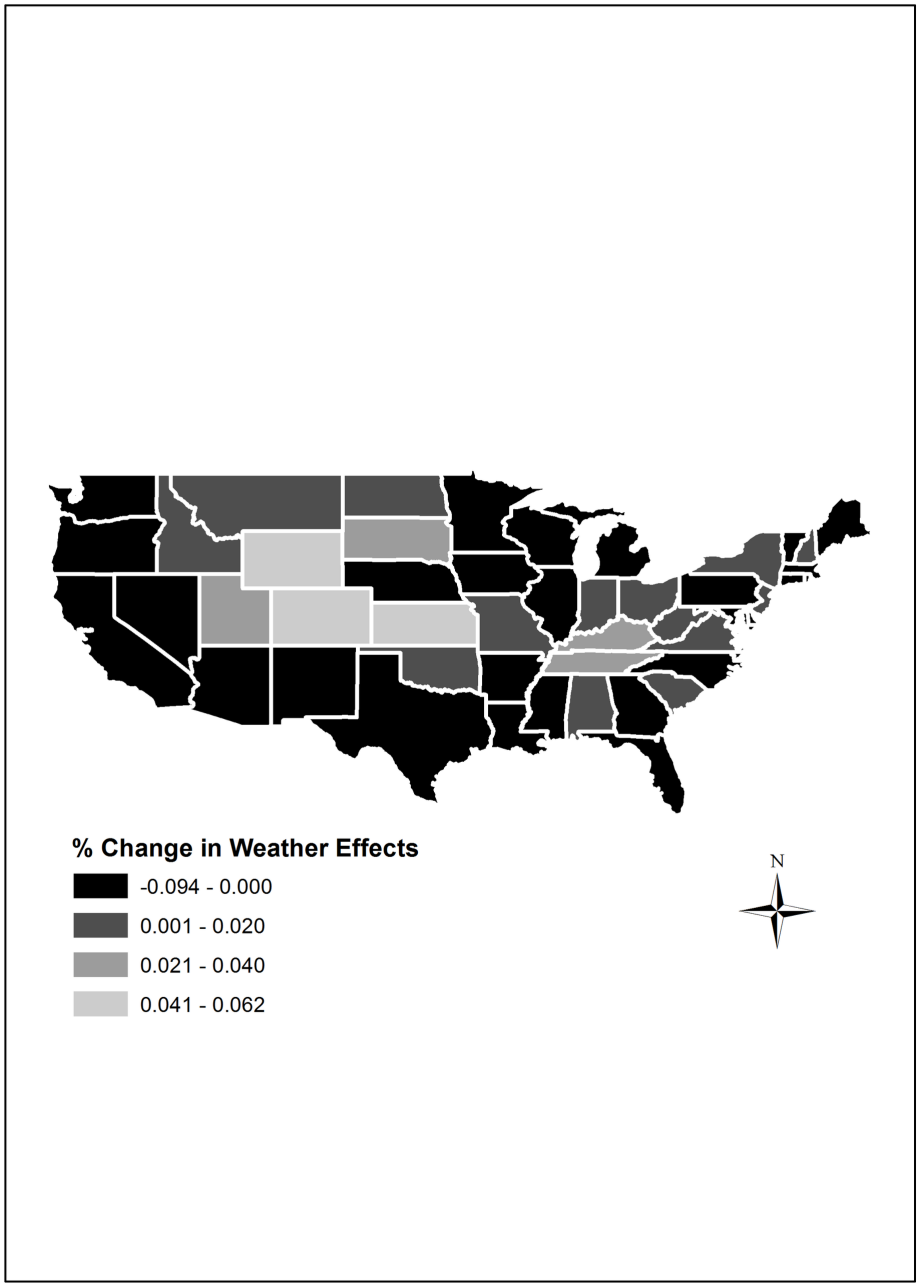
Percentage change in weather effects across the United States, 1960–2004.

The key research objective of this article is to establish the impact of weather variation in year-to-year fluctuations in TFP growth in the U.S. The results (see Table 3) reveal that weather effects, as captured by OEI, on average account for a negligible 0.012% decline in annual TFP growth across the United States. Changes in the OEI over time within any given state are a measure of the effect of changes in weather in that state. This is because environmental features captured by the state-level fixed effects are time-invariant. On the other hand, comparisons of OEI across states in any given period are a measure of both state-level fixed effects and weather in that period. Based on this, we find evidence of considerable variation across states as reported in the sixth column of Table 3. In general, weather effects contributed negatively to TFP growth across the Pacific region, the Southwest, parts of the Midwest, and the Northeast; while contributing positively in the Northern Plains and Mountain states as illustrated in Fig 7.

Though the last year of this analysis is 2004, the evidence indicates that weather variability has continued to increase over the last decade. The third National Climate Assessment report [1] singles out recent droughts and heavy precipitation as the biggest threats to the U.S. agricultural sector, and notes that crop and livestock productivity will be negatively impacted as critical temperature and precipitation thresholds are met and exceeded. Malcolm et al. [35] observe that the U.S. agricultural sector’s adaptive capacity may offset some of the negative impacts of the changing climate in the short-run, and that warming may be beneficial to agricultural production as a result of a longer growing season, albeit with strong regional differences. Our general findings are consistent with these reports. It is important to point out that though some states appear to have experienced positive climatic effects, the IPCC [36] report argues that any benefits associated with climate change might dissipate as the agricultural sector’s adaptive capacity is overwhelmed due to critical temperature and precipitation thresholds being surpassed.

## Concluding remarks

This article builds upon previous studies that have analyzed total factor productivity (TFP) trends in U.S. agriculture [27,29,33]. We extend these recent analyses by explicitly introducing weather variables into a stochastic production frontier model. Consequently, we provide new results concerning TFP growth in U.S. agriculture in a way that accounts for the effect of weather variation. Another salient contribution of this paper is the use of the general TFP index that was proposed in O’Donnell [20], which satisfies a suite of economically relevant axioms from index theory and makes it possible to derive a complete decomposition of TFP change.

The primary objective of this article is to evaluate the impact of weather variation on TFP growth. We find that U.S. agricultural TFP growth averaged 1.56% per annum between 1960 and 2004. The key driver behind this TFP growth rate was technological progress, which averaged 1.21% per annum. Moreover, our results reveal that, on average, weather effects were responsible for a negative, albeit negligible, impact on TFP growth, contributing to a 0.012% decline in annual TFP change. Notwithstanding, it is important to emphasize the substantial heterogeneity in the role of weather effects on TFP change across states and over time. The other components, output-oriented technical efficiency and output-oriented scale efficiency, contributed 0.057% and -0.085% to per annum TFP growth, respectively. A state-by-state analysis reveals wide-ranging TFP changes.

The ability to respond appropriately and in a timely fashion to the adverse effects of changing configurations in weather is expected to have a significant impact on future agricultural productivity and food security. Hence, decomposing TFP indexes is crucial from a policy perspective in order to design appropriate responses. The U.S. Federal and State governments have instituted multiple policy mechanisms (e.g., price supports, input subsidies, and various tax schemes) aimed at raising productivity and farm incomes [60,61]. However, as climate variability becomes an increasing burden [36], future policy interventions should focus on promoting mitigation and adaptation activities to cope with such challenges. Public support should be directed to two priority areas. One such area is investments in research and development as well as in adaptive measures. This is informed by our results indicating that technological change has had a positive effect on agricultural output, as well as being the primary driver behind TFP growth. We also propose that investments be directed towards management practices that promote increased farm efficiency. This may take the form of tailored educational and training programs focusing on best management practices that allow for the large spatial variation in weather and agro-ecological factors. This latter recommendation stems from our finding that output-oriented technical efficiency has a substantial impact on TFP growth.

Finally, future research should adopt a micro-level productivity approach to capture salient characteristics within individual states, including detailed analysis for different crop and livestock systems. Bearing in mind the continuing expansion in satellite and remote sensing capabilities, it should become cost effective to generate localized climatic information that can then be coupled with micro-level input-output data. This combined information would significantly enhance the analysis of the interaction between productivity and changing configurations in weather.

## Supporting information

S1 File Dataset used in estimating stochastic production frontier model and decomposing U.S. total factor productivity, 1960–2004. (ZIP)
